# Experiences of psychotherapists working with refugees in Germany: a qualitative study

**DOI:** 10.1186/s12888-020-02996-0

**Published:** 2020-12-11

**Authors:** Baye Berihun Asfaw, Claudia Beiersmann, Verena Keck, Christoph Nikendei, Janine Benson-Martin, Inken Schütt, Julia Lohmann

**Affiliations:** 1 Department of Psychology, College of Social Sciences and Humanities, University of Gonder, P.O. Box: 196, Gonder, Ethiopia; 2 Heidelberg Institute of Global Health, Heidelberg University Hospital, Heidelberg, Germany; 3 Department of Social and Cultural Anthropology, Goethe University Frankfurt/Main, Frankfurt, Germany; 4 Department of General Internal Medicine and Psychosomatics, University Hospital Heidelberg, Heidelberg, Germany; 5 Gesundheitsamt Enzkreis, Pforzheim, Germany; 6 Independent Psychotherapist, Krefeld, Germany; 7 Department of Global Health and Development, London School of Hygiene and Tropical Medicine, London, UK

**Keywords:** Mental health, Refugees, Psychotherapy, Germany, Explanatory models, Communication, Expectations

## Abstract

**Background:**

Despite a high burden of mental health problems among refugees, there is limited knowledge about effective mental health care provision for this group. Although substantial efforts in understanding the complexity of cross-cultural psychotherapy – which in the context of this study we use to refer to therapy with client and therapist of different cultural backgrounds – have been made, there remains a dearth of research exploring barriers for effective cross-cultural psychotherapy. This study aimed at narrowing this gap in knowledge by exploring major challenges encountered by psychotherapists in cross-cultural psychotherapy and strategies which have proven useful in overcoming such challenges.

**Methods:**

We employed a qualitative study design, conducting semi-structured in-depth interviews with 10 purposely selected psychotherapists working with refugees in Germany. Respondents were from varying theoretical background and had varying levels of experience. Data were analyzed using a thematic approach, following a mix of deductive and inductive coding.

**Results:**

Respondents reported three main challenges in their cross-cultural practice: different or unrealistic expectations of clients towards what psychotherapy would offer them; challenges grounded in different illness explanatory models; and communication challenges. In dealing with these challenges, respondents recommended psychoeducation to overcome issues related to problematic expectations towards psychotherapy; “imagining the real”, identifying “counter magic” and other client-appropriate resources to deal with issues related to clients’ foreign illness attributions; and translators in dealing with communication barriers, though the latter not univocally.

**Conclusions:**

Results show that psychotherapy with refugees can be very successful, at least from the psychotherapist perspective, but also poses significant challenges. Our findings underline the importance of developing, testing, and institutionalizing structured and structural approaches to training psychotherapists in cross-cultural therapy at scale, to accommodate the rising mental health care need of refugees as a client group.

## Background

In recent years, migration has posed substantial challenges to European health systems [1, 2]. Between 2010 and 2019 about 79.9 million people were forcibly displaced worldwide, of which 9.2 million people from different parts of the world emigrated to the European Union as first-time asylum seekers applying for international protection. Germany as one of the top 5 refugee and asylum seeker host countries registered 165,938 new asylum claims in 2019 alone [3].

Asylum seekers and refugees are exposed to various traumatic events pre-, peri-, or post-migration, which put them at high risk to develop mental health issues [4, 5]. Recent reviews on the prevalence of mental health issues among young and adult refugees and asylum seekers in Europe reported higher prevalences of post-traumatic stress disorder, anxiety disorders, depression, and other mental health issues compared to the general population [6–8], although with substantial variation by origin and other factors.

This emerging client group and their health care needs challenge existing care systems in host countries, including Germany. Although the European Charter of Fundamental Rights stipulates that all migrants and refugees should be entitles to at least emergency and essential primary health care, including psychotherapy [9], challenges in accessing care and particularly mental health care are manifold and have been relatively well documented. Key issues include systemic factors such as legal restrictions [10–14]; provider-level barriers such as lack of awareness of regulations, reluctance related to cultural differences and competence, and capacity constraints particularly for psychotherapy [15–19]; and individual-level factors such as lack of trust, unfamiliarity, and irritation with the host country health system, financial access to care in systems which are not free of charge at point of use but rather rely on health insurance or reimbursements, and general perceived discrimination [17, 18, 20–22]. The extent to which these factors limit refugees’ actual access to mental health care are underlined by a recent study showing that in a major German refugee registration and reception center, not one of the newly arrived refugees referred to psychotherapy had received outpatient psychotherapeutic treatment within 3 months of their referral [23].

This is particularly unfortunate as studies show that both established therapeutic methods and approaches adapted or specifically developed to working with refugee clients can be effective in treating their mental health issues [24–29], despite fundamental criticism of “fitting” refugees within Western concepts and classifications of mental illness brought forward by some authors [30].

However, this does not mean that no challenges in working with this group exist. In contrast to the fairly elaborate literature on barriers in access to psychotherapy and on the general effectiveness of psychotherapy with refugees, little research has been done on how psychotherapists, who often are not specifically trained in working with refugee clients [28], experience the cross-cultural psychotherapeutic process, which we define as a therapeutic encounter between a psychotherapist and a client of different cultural backgrounds. The little available research highlights issues such as communication difficulties, differences in illness attribution belief systems, differences in expectations towards treatment, and issues related to trust as main challenges [15, 26, 31–33].

The study therefore aims to contribute to this yet slim body of evidence by exploring psychotherapists’ experiences in conducting psychotherapy with refugees and asylum seekers. In particular, the study aimed to understand the challenges psychotherapists experience in their outpatient practice with this client group, as well as the explicit and implicit strategies psychotherapists use in dealing with these challenges. In doing so, we hope to contribute to the repository of good practices and the development of approaches which are not only effective, but also easy and comfortable for both clients and psychotherapists.

## Methods

### Study context

The study was conducted in Germany, which, among the top refugee and asylum seeker host countries in the world, admitted a total of about 1.1 million asylum seekers between 2010 and 2019 and received a total of 165,938 new asylum claims in 2019 alone [3]. Refugees are entitled to treatment for different acute conditions including psychotherapy for a period of 15 months after being granted asylum seeker status. This includes translation services if required. In practice, however, administrative hurdles and significant supply side challenges to meeting demand (including general capacity and specific reluctance of psychotherapists to work with refugees) limit the extent to which existing needs can be met [34]. Once the initial 15-month period has been exceeded, decisions on whether and by whom costs are covered are made on a case-by-case basis. Once asylum seekers are granted asylum, they enter the statutory health insurance system, and its regulations apply, including an entitlement to short-term psychotherapy in case of diagnosis, but not to translation services [35]. However, general supply-side challenges limit the extent to which demand for psychotherapy can be met.

### Study design and sample

We conducted a qualitative study to explore psychotherapists’ lived experiences in working with refugees or asylum seekers, both adult and minor. The sample included licensed psychotherapists practicing in Germany who had completed treatment with at least one refugee or asylum seeker (irrespective of client age) and who were comfortable being interviewed in English, as interviews were carried out by the first author who is not fluent in German. Respondents were recruited through the authors’ professional networks and through the German Association of Psychosocial Centers for Refugees and Torture Victims, which hosts a list of therapists offering psychotherapeutic services for refugees and asylum seekers.

Table 1 provides details on the study sample. All participants were licensed psychotherapists, most with a theoretical background in Cognitive Behavior Therapy or Psychoanalysis, and worked exclusively on an outpatient basis. Most participants were female, and most had an educational background in psychology. Professional experience ranged from 2 to 35 years, and all but one indicated having treated at least 10 refugee or asylum seeker clients, some substantially more. 6 out of 10 respondents reported to have lived abroad for some time in their lives, while the remaining 4 respondents did not have any own international experience beyond travel on holidays. Only one of the respondents reported having had specific training in cross-cultural therapy and/or working with refugee clients.

**Table 1:** Demographic characteristics of participants

ID	Gender	Educational background	Theoretical background	Experience in years	No of treated refugees / Asylum seekers
IN1	Female	Medicine	Psychoanalysis	7	> 10
IN2	Female	Psychology	Humanistic Psychology	35	> 10
IN3	Female	Psychology	Psychoanalysis	4	> 9
IN4	Female	Medicine	Psychoanalysis	2	> 10
IN5	Female	Psychology	Cognitive Behavior Therapy	10	> 10
IN6	Male	Psychology	Cognitive Behavior Therapy	2	> 10
IN7	Female	Psychology	Cognitive Behavior Therapy	5	> 10
IN8	Female	Psychology	Cognitive Behavior Therapy	5	> 10
IN9	Male	Psychology	Cognitive Behavior Therapy	32	> 10
IN10	Female	Psychology	Psychoanalysis	10	> 10

### Data collection procedure

The study was approved by the Research Ethics Committee of Heidelberg University (protocol number S-324/2018). All respondents had given written informed consent to participation and to having their anonymized information used in peer-reviewed publications.

Data was collected through in-depth interviews by the first author (not fully fluent in German) in English language. The interviewing process showed that all participants were at ease at expressing themselves in English, allowing us to obtain rich data. Preference was given to conducting interviews face-to-face, but this was only possible for 4 respondents who resided in reasonable distance from the interviewer. All other interviews were conducted via phone.

Interviews were conducted along a semi-structured interview guide developed for the purpose of the study (see Additional Files 1), which included questions to explore respondents’ cross-cultural expertise and experiences as background information to situate their narratives; questions regarding their experiences with refugee and asylum seeker clients and the challenges they had encountered in their therapeutic practice to date; and questions relating to the strategies they had successfully used in their practice to overcome these challenges. The interview guide was rigorously reviewed by the author team and other experts in the study area within the authors’ network (from various fields including psychology, anthropology, psychiatry and transcultural psychiatry) to increase its construct validity.

Interview length varied between 47 min and 1 h and 42 min, depending on the amount and depth of information volunteered by the respondent. All interviews were audio recorded and then verbatim transcribed.

To further ensure trustworthiness of the collected data, respondents were offered to review their transcripts to ensure accurate representation of the intended communicated information [36]. Two respondents took up the offer but did not request any modifications.

### Data analysis

We employed a thematic approach to analysis [37], conducted by the first author and triangulated by the last author. Following familiarization with the material, transcripts were coded using a mix of deductive and inductive coding, where an initial codebook was developed based on the interview guide and themes having emerged in previous research, which was then expanded as we proceeded through the material. The resulting coded material was analyzed by theme across participants. NVivo was used to facilitate the process of coding, organizing, and analyzing the material. Emerging results and initial interpretations were then discussed to arrive at a joint interpretation.

## Results

In the following, we first present the main challenges in working with refugee clients reported by participants, and then the strategies they have found useful in dealing with these challenges.

### Challenges

Respondents identified three groups of main challenges, namely different or unrealistic expectations of clients towards what psychotherapy would offer them; challenges grounded in different illness explanatory models (belief systems); and communication challenges.

#### Expectations towards psychotherapy

Respondents reported various incidences in which they perceived their clients as lacking understanding and knowledge about how psychotherapy works, and how this created confusion, frustration or disappointment, thereby challenging the therapeutic process.

*The idea of psychotherapy in this Western way can be pretty alien to some of them. I mean, you go to someone you do not know, and you are supposed to talk about yourself? What good is that supposed to do? (IN2).*

Respondents differentiated between ‘unrealistic’ and ‘different’ expectations towards psychotherapy. *Unrealistic expectations* refer to clients holding the idea that psychotherapists have answers to all their questions and can ease all their problems. For instance, one respondent described how “*they would expect that the djinn disappears or that I have more knowledge about those traditional healing methods” (IN8).* Several participants described such unrealistic expectations as the most frustrating and challenging aspect regarding their work with refugees. *Different expectations*, in contrast, refer to client expectations about services or benefits which are not per se unrealistic, but not within the psychotherapists’ power or mandate. Almost all respondents spoke about how they have had clients expecting them to give them or help them with residence permits, for instance.

*Sometimes we reach the conclusion that we cannot really help the person because maybe he does not really understand the concept or expects something we cannot offer. For example, they just ask us to help them get their [asylum] claim accepted. We are not the federal office, so we tell them we cannot help them. (IN6).*

#### Cultural challenges – differences in explanatory models (belief systems)

Seven respondents further spoke about having had clients with belief systems or narratives about their illnesses which they perceived as strange and difficult to grasp. One respondent spoke of *fairy tale* when recounting her client’s illness attribution beliefs. Respondents admitted to difficulties in working with refugee clients who believed that demons, spirits, or djinns are the cause of their suffering, and who attributed their illness to being cursed or otherwise subjected to magic.

*They might have a very different model. A sense of “someone used magic against me, and that is why I have this symptom”. Very difficult to work with. (IN2).*

*This is a form of magic thinking in my opinion. If they have dreams, often, they think of djinns and so on. (IN1).*

*Sometimes when people come with demons and ghosts, that is where I struggle a lot. If somebody says I have a demon or a ghost in my stomach, … I do not understand. (IN8).*

#### Communication-related issues

Communication was cited as a challenge by all respondents, which they perceived not only as a hassle, but which seriously impacted on the progress they made with their clients.

*There is a language issue and often when people who come from other countries, not everyone speaks English. That is the challenge we pay attention to. What is the meaning of these words for that person? Do I understand the meaning correctly? (IN2).*

Several participants stressed the importance of communication in building trust and good working relationships. They described how they have experienced this process of building trust and mutual understanding as substantially hindered by the communication barrier, thereby slowing down therapeutic progress.

*The challenge is to have a communication where there is understanding. Like giving them the feeling through communication and eye contact that they can talk about it, the problem. Otherwise, they decide ‘Ok, I do not feel good about this’, and then they will not talk. (IN8).*

### Strategies used by participants to overcome the challenges

Respondents’ strategies in dealing with the aforementioned challenges and barriers are presented in the following. In summary, respondents recommended psychoeducation to overcome issues related to problematic expectations towards psychotherapy; *imagining the real*, identifying *countermagic* and other client-appropriate resources to deal with issues related clients’ foreign illness attributions; and translators in dealing with communication barriers, though with caveats.

#### Dealing with expectations - Psychoeducation

Unrealistic and different expectations of clients about what psychotherapy can do for them were mentioned as one of the key challenges, with the potential to undermine trust and client openness early into the therapeutic relationship. Participants assumed that such expectations were grounded in a lack of knowledge, and thus emphasized the importance of taking the time to inform clients about the role of psychotherapists and what psychotherapy can and cannot do for them in straightforward language. In that context, participants underlined the importance of being adequately informed about the asylum procedures and the ability to refer clients to the responsible bodies as an essential strategy for their work with their refugees, even if in principle not their responsibility.

*We try to explain to them that it is not our role, that we are independent and that we are not deciding [about residence permits]. This is something we have to do very often because the expectations are very different. (IN6).*

#### Dealing with culture-related challenges – “Imagining the real”

In regards to dealing with clients’ divergent belief systems regarding their illness, the most frequently cited strategy was open-mindedness. However, participants underlined how challenging it is to remain unbiased and open-minded to different belief systems. IN2 used the term *imagining the real* to refer to the process of putting oneself into the position of the client and trying to imagine what it means to feel and believe like the client, with the aim of a better understanding of the gist of the message communicated by clients in regards to their illness attribution. Other respondents similarly underlined the importance of actively remaining aware, open and neutral to even the most foreign beliefs and perspectives, and of remaining detached from the influence of one’s belief system.

*To leave back your own way of thinking and living and to only see the world of the patient. You should see the world through the eyes of the patient, and at the same time, compare it with their reality. (IN1).*

#### Dealing with culture-related challenges – Identifying *countermagic* and other resources in therapy

Several respondents underlined that arguing against the clients’ belief system is neither appropriate nor helpful. Rather, respondents reported having made good experiences with helping clients explore what could be done in light of their beliefs of the origin of their problems, even if for instance through unconventional ‘cultural rituals’ to help them recover from *inflictions of the spirits*. One respondent referred to this as helping clients identify *countermagic*, so practices, rituals, or other things or actions likely to improve wellbeing within the client’s belief system. Most of the participants agreed that being open to different and foreign perspectives is inextricably tied and prerequisite to finding clients’ cultural solutions to their own problems. Depending on the identified *countermagic*, it could be used in therapy sessions through simulation, or therapists might encourage clients to pursue the *countermagic* outside of the therapy session.

*To imagine their world well enough to maybe say, ‘Well, what could be countermagic?’ Because if someone is firmly grounded in a belief system, in my experience it does not make any sense to say, ‘It is not magic, let me explain to you what it probably is!’ (IN2).*

*I am sure that there are some things I do the healer cannot do, and I am sure that there are some things that the healer can do that I cannot do. I will tell them okay, I will help you with [your problem], but if you think that this is not sufficient, also try for yourself to find a healer. (IN8).*

The most cited *countermagic* or therapeutic resource was religion. All participants emphasized spirituality and the concept of religion as an essential resource that they actively explored or at least remained conscious of in the therapeutic process, in light of their experience of the importance and impact of religion in their clients’ lives.

#### Dealing with communication-related challenges - translators as solutions

Strategies reported by participants in their effort to deal with language problems differed substantially. Six of the participants expressed a strong belief that translators are solutions for language barriers, and they reported consistent use of translators.

*In the beginning we all have translators, and I think this is very helpful for both of us. Because they know the country, the culture, and what is common. And as a psychotherapist, the first step is to understand the person in her culture. And translators help us with that. (IN7).*

Other participants expressed an opposite opinion, believing that the use of translators has more challenges than benefits. Specifically, they described how while overcoming the issue of language per se, the translator was often a challenge as they perceived them to not accurately transport the clients’ feelings and experiences, but rather adding their own feelings and interpretations to the translation.

*Sometimes translators want to do translating in the sense that they know everything. They do not just translate the question, they do not make the voice of the patient be heard, but they use the situation to make it their own stage. They try to act as therapists themselves sometimes. (IN10).*

Thus, they rather recommended working without translators and using non-verbal approaches like sand play, where clients are asked to express their thoughts and feelings using forms and shapes in the sand, drawings, body language, and other non-verbal communication, especially in working with young refugees.

*You can put them [the feelings, experiences] in the sand and make a picture. This picture which the people are making is spontaneous. It is an expression of their soul situation. And we see in the refugee patients that they very often start with making a picture from the trauma they experience. Maybe we see much blood, destroyed houses and so on. This picture will be some sort of release for their soul because they can find a way to express their feelings. (IN1).*

## Discussion

Our study contributes to filling a gap in the current literature by exploring psychotherapists’ experiences, challenges, and success strategies in their work with refugee clients, an area not well researched in the otherwise fairly well-explored field of refugee mental health. Respondents identified three main challenges: a mismatch between refugee clients’ expectations towards psychotherapy, and what they could realistically do for them; diverging belief systems as to the etiology of the clients’ problems; and communication barriers. This largely echoes the few existing studies on psychotherapists’ experiences [15, 26, 31–33]. In response to these challenges, respondents reported good experiences with spending time educating patients on the role and process of psychotherapy; with trying to understand, remaining open to, and actively utilizing resources aligned with the client’s belief system; and in part also with the use of translators, although almost half of the respondents preferred using non-verbal communication over the use of translators.

As reviewed in the introduction, more and more research emerges on the effectiveness of therapeutic interventions targeted specifically at refugee clients [24–29], based at least in part on a large body of literature about illness attribution from the field of global mental health [e.g. 38–40]. Interestingly, such specific therapeutic approaches to working with refugee clients were not mentioned at all by the study respondents. Similarly, common tools such as cultural formulation instruments [41] which aid in the active exploration of clients’ belief systems were not mentioned by name, despite their function and functioning being described as commonly used in practice. Almost half of the participants spoke very critical about the use of translators, despite robust evidence that psychotherapy can work well with and even benefit from well-trained translators [42–44].

This underlines perhaps the biggest problem in addressing mental health of refugees and asylum seekers: Although substantial influx of refugees has long become a reality and elevated mental health care needs are well known, and although tools to adequately do so are well established in principle, health and social security systems have not only failed in enabling de facto access to mental health care, but also in preparing health system actors for the challenges they face in working with this new client group.

All but one of our participants had never received any formal training in working with this client group, despite many having treated a substantial number of refugee clients. Although it remains unclear whether due to a lack in offer or rather in uptake thereof, this mirrors observations by others [e.g. 28, 45]. Similarly, our findings imply that well-trained translators are in short supply. Although practical guidance is widely available [e.g. 46] and some formal training programs exist [e.g. 47], tangible evidence on their effectiveness is largely lacking. In the absence of formal regulations and large-scale training offers, it is largely in psychotherapists’ own responsibility and initiative to gather information and strengthen their cross-cultural therapy skills.

On a positive note, our findings imply that psychotherapist seem to “muddle through” quite well in everyday practice, as much of what they describe in “lay terms” mirrors common recommendations in the expert literature well. While respondent accounts illustrate their motivation, resourcefulness, and intuition, they also underline their struggles. In describing their strategies to overcome encountered challenges, respondents indirectly underlined a major risk of low-quality cross-cultural psychotherapy: inadvertently propagating clients’ mental health issues and contributing to rather than alleviating the many detrimental post-flight stressors experienced by refugees [48], by frustrating their – albeit misguided – expectations, or by being closed and judgmental towards clients’ foreign beliefs and illness attributions, thereby betraying their trust.

### Study limitations and recommendations for future research

Despite being one of few studies attempting to document psychotherapists’ perspectives on the challenges of working with refugees, our study should be read in light of its limitations. First, the small sample size limits its representativeness. Second, interviews were conducted in English by the first author who is not fluent in German. While all respondents appeared comfortable in expressing themselves in English, this might have biased our sample towards culturally more aware, multi-lingual participants. We further employed a mix of face-to-face and phone interviews. Although the data does not suggest so, we cannot exclude that differential response biases were at play. Further, we asked respondents explicitly to limit their accounts to experiences with refugee clients. However, many respondents have worked with a wide range of clients also including such with a migration, but not a refugee background. We cannot exclude that experience with these client groups are also reflected in the presented results.

Our sample size and interviewing approach unfortunately did not allow for a specific analysis of differences between therapists treating adults and children and adolescents. This will be an interesting area to explore in future research. Further interesting areas for future research include a more in-depth exploration of psychotherapists’ stereotypes and preconceptions towards refugees and their illness attributions, and an exploration of the role of other parties involved in the asylum seeking process (e.g. government officials, refugee center workers) in shaping refugees’ mental health, healthcare seeking, and treatment experience.

## Conclusions

Respondents reported various challenges in working with refugee clients. Despite describing inspired strategies in overcoming these challenges, they underlined the difficulties entailed in working with this emerging client group. Our findings underline the importance of developing, testing, and institutionalizing approaches to training psychotherapists in cross-cultural therapy at scale, given the rising importance of refugees as a client group. Structural solutions to the “refugee mental health crisis”, not only in relation to access to care, but also in relation to adequately preparing the mental health care workforce in working with this emerging client group, are urgently necessary.

## Supplementary Information

**Additional file 1.**

## Data Availability

The datasets generated and analyzed in the current study are not publicly available so as not to compromise respondent confidentiality, as individuals might be identifiable by what they said even in the absence of names or other identifying information. However, they might be available from the corresponding author on reasonable request.

## References

[1] Carballo M, Hargreaves S, Gudumac I, Maclean EC (2017). Evolving migrant crisis in Europe: implications for health systems. Lancet Glob Health.

[2] Humphris R, Bradby H, McQueen D (2017). Health status of refugees and asylum seekers in Europe. Oxford research encyclopedia of global public health.

[3] Bundesamt für Migration und Flüchtlinge. [The federal office for migration and refugees in numbers 2019 - module asylum.] 2020. https://www.bamf.de/SharedDocs/Anlagen/DE/Statistik/BundesamtinZahlen/bundesamt-in-zahlen-2019-asyl.html. Accessed 26 Aug 2020.

[4] Rousseau C, Frounfelker RL (2019). Mental health needs and services for migrants: an overview for primary care providers. J Travel Med.

[5] Kirmayer LJ, Narasiah L, Munoz M, Rashid M, Ryder AG, Guzder J (2011). Common mental health problems in immigrants and refugees: general approach in primary care. CMAJ..

[6] Sommer I, Kien C, Faustmann A, Gibson L, Schneider M, Krczal E (2018). Prevalence of mental disorders in young refugees and asylum-seekers in European countries. Eur J Pub Health.

[7] Blackmore R, Gray KM, Boyle JA, Fazel M, Ranasinha S, Fitzgerald G (2020). Systematic review and meta-analysis: the prevalence of mental illness in child and adolescent refugees and asylum seekers. J Am Acad Child Adolesc Psychiatry.

[8] Brandt L, Henssler J, Mueller M, Wall S, Gabel D, Heinz A (2019). Risk of psychosis among refugees: a systematic review and meta-analysis. JAMA Psychiatry.

[9] European Union (2011). European agency for fundamental rights. fundamental rights of migrants in an irregular situation in the European Union.

[10] Norredam M, Mygind A, Krasnik A (2006). Access to health care for asylum seekers in the European Union. A comparative study. Eur J Pub Health.

[11] O’Donnell CA, Burns N, Mair FS, Dowrick C, Clissmann C, van den Muijsenbergh M (2016). Reducing the health care burden for marginalised migrants: the potential role for primary care in Europe. Health Policy.

[12] Bell P, Zech E (2009). Access to mental health for asylum seekers in the European Union. An analysis of disparities between legal rights and reality. Arch Public Health.

[13] Hadgkiss EJ, Renzaho AM (2014). The physical health status, service utilisation and barriers to accessing care for asylum seekers residing in the community: a systematic review of the literature. Aust Health Rev.

[14] Winkler JG, Brandl EJ, Bretz HJ, Heinz A, Schouler-Ocak M (2019). The influence of residence status on psychiatric symptom load of asylum seekers in Germany. Psychiatr Prax.

[15] Suphanchaimat R, Kantamaturapoj K, Putthasri W, Prakongsai P (2015). Challenges in the provision of healthcare services for migrants: a systematic review through providers’ lens. BMC Health Serv Res.

[16] Lindenmeyer A, Redwood S, Griffith L, Teladia Z, Phillimore J (2016). Experiences of primary care professionals providing healthcare to recently arrived migrants: a qualitative study. BMJ Open.

[17] Straßmayr C, Matanov A, Priebe S, Barros H, Canavan R, Díaz-Olalla JM (2012). Mental health care for irregular migrants in Europe: barriers and how they are overcome. BMC Public Health.

[18] Giacco D, Matanov A, Priebe S (2014). Providing mental healthcare to immigrants: current challenges and new strategies. Curr Opin Psychiatr.

[19] Satinsky E, Fuhr DC, Woodward A, Sondorp E, Roberts B (2019). Mental health care utilisation and access among refugees and asylum seekers in Europe: a systematic review. Health Policy.

[20] Kang C, Tomkow L, Farrington R (2019). Access to primary health care for asylum seekers and refugees: a qualitative study of service user experiences in the UK. Br J Gen Pract.

[21] O'Donnell CA, Higgins M, Chauhan R, Mullen K (2007). "they think we're OK and we know we're not". A qualitative study of asylum seekers' access, knowledge and views to health care in the UK. BMC Health Serv Res.

[22] O'Donnell CA, Higgins M, Chauhan R, Mullen K (2008). Asylum seekers' expectations of and trust in general practice: a qualitative study. Br J Gen Pract.

[23] Nikendei C, Kindermann D, Brandenburg H, Derreza-Greeven C, Zeyher V, Junne F (2019). Forced migrants’ access to psychiatric and psychotherapeutic care: a follow-up study after transfer from a state registration- and reception-center in Germany. Health Policy.

[24] Rathod S, Gega L, Degnan A, Pikard J, Khan T, Husain N (2018). The current status of culturally adapted mental health interventions: a practice-focused review of meta-analyses. Neuropsychiatr Dis Treat.

[25] Slobodin O, De Jong JT (2015). Mental health interventions for traumatized asylum seekers and refugees: what do we know about their efficacy?. Int J Soc Psychiatry.

[26] Gruner D, Magwood O, Bair L, Duff L, Adel S, Pottie K (2020). Understanding supporting and hindering factors in community-based psychotherapy for refugees: a realist-informed systematic review. Int J Environ Res Public Health.

[27] Lambert JE, Alhassoon OM (2015). Trauma-focused therapy for refugees: meta-analytic findings. J Couns Psychol.

[28] Munz D, Melcop N (2018). The psychotherapeutic care of refugees in Europe: treatment needs, delivery reality and recommendations for action. Eur J Psychotraumatol.

[29] Kip A, Priebe S, Holling H, Morina N (2020). Psychological interventions for posttraumatic stress disorder and depression in refugees: a meta-analysis of randomized controlled trials. Clin Psychol Psychother.

[30] Watters C (2001). Emerging paradigms in the mental health care of refugees. Soc Sci Med.

[31] Sandhu S, Bjerre NV, Dauvrin M, Dias S, Gaddini A, Greacen T (2013). Experiences with treating immigrants. Soc Psychiatry Psychiatr Epidemiol.

[32] Al-Roubaiy NS, Owen-Pugh V, Wheeler S (2017). Iraqi refugee men’s experiences of psychotherapy: clinical implications and the proposal of a pluralistic model. Brit J Guid Couns.

[33] Kiselev N, Morina N, Schick M, Watzke B, Schnyder U, Pfaltz MC (2020). Barriers to access to outpatient mental health care for refugees and asylum seekers in Switzerland: the therapist's view. BMC Psychiatry.

[34] Bundesweite Arbeitsgemeinschaft der psychosozialen Zentren für Flüchtlinge und Folteropfer e.V. (BAFF e.V.). [Administrative regulations on the application procedure for psychotherapy according to AsylbLG.] 2018. http://www.baff-zentren.org/wpcontent/uploads/2008/05/Musterverwaltungsvorschrift-f%C3%BCr-Psychotherapien-im-AsylbLG.pdf. Accessed 5 Nov 2019.

[35] Wissenschaftliche Dienste des deutschen Bundestages (2017). Situation report: Interpreters in the context of health care entitlement and cost coverage.

[36] Willig C (2013). Introducing qualitative research in psychology.

[37] Mayring P, Kiegelmann M (2002). Qualitative content analysis: research instrument or mode of interpretation?. The role of the researcher in qualitative psychology.

[38] Kleinman A (1978). Culture, illness, and care. Ann Intern Med.

[39] Patel V (1995). Explanatory models of mental illness in sub-Saharan Africa. Soc Sci Med.

[40] Adewuya AO, Makanjuola RO (2008). Lay beliefs regarding causes of mental illness in Nigeria: pattern and correlates. Soc Psychiatry Psychiatr Epidemiol.

[41] Lewis-Fernández R (2009). Editorial: the cultural formulation. Transcult Psychiatry.

[42] Searight HR, Armock JA (2013). Foreign language interpreters in mental health: a literature review and research agenda. N Am J Psychol.

[43] Hunt X, Swartz L (2016). Psychotherapy with a language interpreter: considerations and cautions for practice. S Afr J Psychol.

[44] Brune M, Eiroá-Orosa FJ, Fischer-Ortman J, Delijaj B, Haasen C (2011). Intermediated communication by interpreters in psychotherapy with traumatized refugees. Int J Cult Ment Health.

[45] von Lersner U, Baschin K, Wormeck I, Mösko MO (2016). Guidelines for trainings in inter−/transcultural competence for psychotherapists. Psychother Psychosom Med Psychol.

[46] http://www.baff-zentren.org/veroeffentlichungen-der-baff/literaturempfehlungen/#psychosoziale-und-medizinische-versorgung-von-fluechtlingen. Accessed 16 Aug 2020.

[47] von Lersner U, Baschin K, Hauptmann N (2019). Evaluating a programme for intercultural competence in psychotherapist training: a pilot study. Clin Psychol Rev.

[48] Li SSY, Liddell BJ, Nickerson A (2016). The relationship between post-migration stress and psychological disorders in refugees and asylum seekers. Curr Psychiatry Rep.

