# Supplementary material for: Experiences of psychotherapists working with refugees in Germany: a qualitative study
Source: BMC Psychiatry. 2020 Dec 11;20:588. doi: 10.1186/s12888-020-02996-0 (PMC7733283; doi:10.1186/s12888-020-02996-0)
Supplement: Supplementary file 1 — Additional file 1. [file 12888_2020_2996_MOESM1_ESM.docx]

**Experiences of psychotherapists working with refugees in Germany: A qualitative study**

**In-depth interview guide**

I want to thank you for taking the time to meet with me today. My name is Baye B. Asfaw, and I would like to talk to you about your experiences as a psychotherapist working with immigrants. The main objective of the study is to assess experiences of Psychotherapists specifically related to working with immigrants. The lessons from this study will widen the knowledge about the practice of Psychotherapy with immigrants.

The interview should take less than an hour. I would like to tape the session because I don’t want to miss any of your comments. Although I will be taking some notes during the session, I can’t possibly write fast enough to get it all down. Is that ok with you? [wait for response] Because we’re on tape, please be sure to speak up so that I don’t miss your comments.

All responses will be kept confidential. This means that your interview responses will only be shared with research team members and we will ensure that any information we include in our report does not identify you as the respondent. Remember, you don’t have to talk about anything you don’t want to and you may end the interview at any time.

Are there any questions about what I have just explained?

Are you willing to participate in this interview?

1. Introduction_______________________________________________________________
2. Would you tell me about yourself and your background as a psychotherapist?

Probes

- Where are you from originally?
- have you lived abroad? If so, where? Its implication to what you are doing as a cross-cultural psychotherapist?
- Educational background
- Therapeutic training and practice
- Years of experience as a therapist in general and with immigrants
- Approximate number of migrant patients so far
- International/intercultural experience in general (Specific training in trauma? Any Intercultural trainings?)
- Anything you would like to add about yourself that you think I should know?

1. Would you tell me a bit about your migrant patients?

Possible Probes

- How do you get your patients? Referrals (self or other?)
- What type of patients in terms of case do you usually work with?
- Background/origin
- Age groups, “gender distribution” ----its implication for your therapy as a challenge
- Common issues for which they are seeking treatment, common diagnoses
- Criteria for accepting patients? German Language proficiency?

1. Core interview______________________________________________________
   1. Migrants Vs German patients
2. Would you tell me how you got into working with immigrant patients?
3. In your experience, what makes working with immigrant patients in general unique than working with German patients?

(If working with both)

Exhaustive Probes based on what comes up

Asking for examples to elaborate more

Do you have a tailored treatment approach only to the needs of immigrants?

1. In your experience, what are commonalities between German and immigrant patients?

Cases?

Illness attribution?

Adherence to treatment?

- 1. Explanatory models

1. How do you assess what your patients’ attribute their illness to?

Would you share what you think about the importance of doing so?

Could you give me some concrete examples?

**Possible Probes**

Have you encountered immigrant patients that attribute mental illnesses to supernatural powers? God, spirits etc.

- What is the most common attribution that most of the immigrants make?
- From your experience, what are the implication of such attributions in therapy- in terms of facilitating or complicating the therapeutic process?
  - - - How? Any examples…How do you deal with such patients?
      - How do you see such explanations in light of your own ideals of the cause of their illnesses? Do you give in to such explanations and try to incorporate them?
      - What meanings do you ascribe to such attributions? (religion, spirits etc)
      - Is there any way you can think of to positively use such illness attributions?

1. Can you think of any (other) reason why Psychotherapy with immigrants might be easier?

How does any similarity between you and the refugees make Psychotherapy easier? As well in light of Gender similarities (between Psychotherapist and patient)

Religion

Migration Background

1. What (else) is challenging about working with immigrant patients? Elaborate with examples

As well in light of Gender similarities (between Psychotherapist and patient)

Religion

Migration Background

1. Any additional strategies you use in your practice with immigrant patients?
2. Based on your experiences, what advice would you give to therapists both working and those who aspire to work with immigrants?
3. **Wrap up__________________________________________________________**
4. What makes working with immigrant clients interesting? What keeps you motivated despite the challenges?

1. Is there anything you would like to add?

Thank You again
